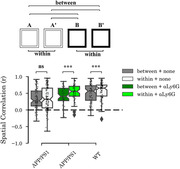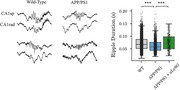# Impaired mechanisms of context encoding in APP/PS1 mice are rescued by increasing cerebral blood flow

**DOI:** 10.1002/alz.088988

**Published:** 2025-01-03

**Authors:** Laura E Berkowitz, Ralitsa Todorova, Dana I Cabus, Jack T Letendre, Natalie Tehrani, Jean‐Luc Shimizu, Xuelai Dong, Azahara Oliva, Antonio Fernandez‐Ruiz, Nozomi Nishimura, Chris B Schaffer

**Affiliations:** ^1^ Cornell University, Ithaca, NY USA

## Abstract

**Background:**

Spatial disorientation is an early symptom of Alzheimer’s disease (AD). The hippocampus creates a cognitive map, wherein cells form firing fields in specific locations within an environment, termed place cells. Critically, place cells remain stable across visits to an environment, but change their firing rate or field location in a different environment. In rodent models of AD‐like pathology, place cells exhibit broadened tuning and altered responses to environmental changes. Additionally, events known as sharp wave ripples (SWRs), coordinate the firing of hippocampal neurons, including place cells, and are disrupted in mouse models of AD. Our lab found that increasing cerebral blood flow (CBF) with anti‐Ly6G antibody treatment improved performance on spatial memory tasks. Here, we investigate the effect of increased CBF on neural mechanisms associated with cognitive map stability across contexts.

**Method:**

We investigated the association between CBF and cognitive map stability in 7‐9‐month‐old APP/PS1 mice and wild‐type controls by recording neural activity in hippocampus area CA1 using 64‐channel silicon probes. Mice were recorded as they explored open field arenas with differing environmental contexts, A and B. Place cells were identified as neurons with significant spatial information in their firing rate maps. Local field potential recordings from pyramidal and stratum radiatum layers of CA1 were used to detect awake‐SWRs (aSWRs).

**Result:**

Place cells from APP/PS1 mice did not exhibit higher place field correlations within contexts (AA' or BB'), as compared to between (AB) contexts, suggesting these different contexts are not differentially encoded (Figure 1). In contrast, wild‐type control mice showed higher within context correlation (Figure 1). APP/PS1 mice also had a reduced rate and duration of aSWR compared to controls (Figure 2). Following treatment with anti‐Ly6G antibodies, both context discrimination by place cells and duration of aSWR increased in APP/PS1 mice (Figure 2).

**Conclusion:**

These results highlight the association between place cell stability and subsequent aSWR dynamics as a potential mechanism contributing to an impaired cognitive map in AD. Importantly, the rescue of behavioral features and aSWR physiological properties following anti‐Ly6G antibody treatment suggests that increasing CBF may be a candidate therapy to mitigate spatial memory impairments in AD.